# Bioimage analysis in deep visual proteomics: Advancing transparency, reproducibility, and FAIR principles

**DOI:** 10.1111/jmi.70145

**Published:** 2026-07-13

**Authors:** Devon Siemes, Angelo Novak, Stephanie Thiebes, Lars Widera, Stephanie Tautges, Hannah Voss, Lars Borgards, Dominik Habermann, Jianxu Chen, Olga Shevchuk, Daniel R. Engel

**Affiliations:** ^1^ Department of Immunodynamics Institute of Experimental Immunology and Imaging University Hospital Essen Essen Germany; ^2^ Department of Biospectroscopy Leibniz‐Institut Für Analytische Wissenschaften ISAS Dortmund Germany

**Keywords:** deep visual proteomics, spatial biology, laser capture microdissection, image registration, open-source software

## Abstract

Deep Visual Proteomics (DVP) combines high‐resolution microscopy, computational instance segmentation, laser capture microdissection (LMD), and ultrasensitive mass spectrometry to enable spatially resolved molecular analysis of defined cellular populations. Within this workflow, LMD plays a central role by physically isolating microscopy‐defined regions of interest for downstream proteomic profiling. However, accurate image‐to‐stage transfer remains challenging due to limitations in imaging resolution, tissue preparation variability, laser cutting parameters, and partially manual alignment procedures. Here, we present an open‐source semi‐automated image‐to‐LMD interoperability framework for DVP that integrates fiducial marker detection, coordinate transformation, and segmentation‐driven contour export for reproducible microdissection. Using neutrophil isolation from murine urinary bladder tissue as a representative use case, we demonstrate robust alignment of computationally defined cellular boundaries with LMD‐guided excision. By improving interoperability, transparency, and workflow standardisation, this framework strengthens the integration of microscopy and spatial proteomics and supports reproducible analysis of spatially defined immune cell populations.

## 1 INTRODUCTION

Deep Visual Proteomics (DVP) combines high‐resolution microscopy and laser capture microdissection (LMD) to isolate cell populations of interest for molecular analysis using liquid chromatography (LC) mass spectrometry (MS). Since its publication in 2022, the emerging technology of DVP has become one of the leading methods in the field of spatial proteomics with bioimage analysis playing a pivotal role in the overall workflow.^1^, ^2^, ^3^, ^4^, ^5^, ^6^ The core workflow involves three tightly coupled steps: (1) segmentation and boundary detection of individual cells from high‐resolution microscopy images, (2) communication of spatial fiducial markers to LMD software, (3) precise excision of selected structures via LMD for downstream proteomic profiling of excised materials. While initial implementations of DVP relied on semi‐manual image preparation and proprietary interfaces for LMD alignment, open access and interoperability frameworks have been lacking. Thus, its success depends on the seamless and automated integration of bioimage analysis and precise LMD procedure. Standardisation, interoperability, and methodological transparency are essential to overcome current bottlenecks. Previous studies have demonstrated the potential of DVP by using BioImage AnalysiS (BIAS) for manual image processing and LMD preparation.^1^, ^4^, ^5^, ^6^, ^7^ However, further automation and adoption of open‐access software for the DVP pipeline are required to improve reproducibility in accordance with the FAIR principles (Findable, Accessible, Interoperable, and Reusable), which provide guidelines for transparent and reproducible data and software handling.^8^, ^9^ While FAIR compliance facilitates software and data discoverability, accessibility, interoperability, and reusability, reproducibility additionally requires comprehensive methodological reporting, software versioning, and parameter transparency. Our workflow addresses FAIR principles through open‐source code availability, version‐controlled releases, persistent identifiers, and standardised XML‐based coordinate exchange, while reproducibility is further supported by detailed metadata and protocol reporting. We provide a re‐implementation of DVP as an open‐access, reproducible, and simple‐to‐use pipeline to gain unprecedented insights into complex cellular adaptations in tissues and disease states. We show how to adapt the DVP workflow to maintain high quality and reproducible data analysis and present an open‐access pipeline of the DVP workflow designed to be simple‐to‐operate, fully reproducible, and exclusively built upon open‐access computational components. The pipeline integrates open bioimage analysis tools for segmentation and annotation, standardised coordinate export for LMD instruments, and automated preparation steps that minimise user intervention. Our workflow adheres to FAIR principles by (i) providing version‐controlled open‐source codes @Github, WorkflowHub and Zenodo (Findable and Accessible), (ii) using standardised XML‐based coordinate exchange formats (Interoperable), and (iii) documenting segmentation parameters, software versions, and laser settings to ensure reproducibility (Reusable). Furthermore, we outline practical considerations for maintaining high data quality across imaging, segmentation, coordinate transfer, and microdissection, and discuss how the workflow can be adapted and extended for diverse tissues and experimental conditions. By lowering technical barriers and ensuring FAIR compliance, this open‐source re‐implementation aims to make DVP broadly usable while preserving the methodological rigour required for quantitative spatial proteomics.

## 2 RESULTS AND DISCUSSION

### 2.1 Overview of the DVP pipeline and the open‐source re‐implementation

DVP is the measurement of the proteome of precisely cut single cells or micro‐tissue regions from complex environment (Figure 1A). This work outlines the core principles underlying the imaging‐related aspects of DVP, emphasising the integration of high‐resolution microscopy, computational segmentation, and LMD for subsequent spatially resolved proteomics. Typically, careful adaptations are required to accommodate tissue type, cellular density, and morphological heterogeneity of the tissue microenvironment. Here, we provide a semi‐automated pipeline, particularly for cell segmentation, boundary detection, and registration of images through fiducial markers. By optimising and automating these components, we demonstrate precise and reproducible excision of neutrophils as a representative use case to study the cellular and molecular changes of this cell population in tissues. These refinements provide a practical framework for applying DVP to other immune or spatially restricted cell populations in a reproducible and semi‐automated manner.

**FIGURE 1:**
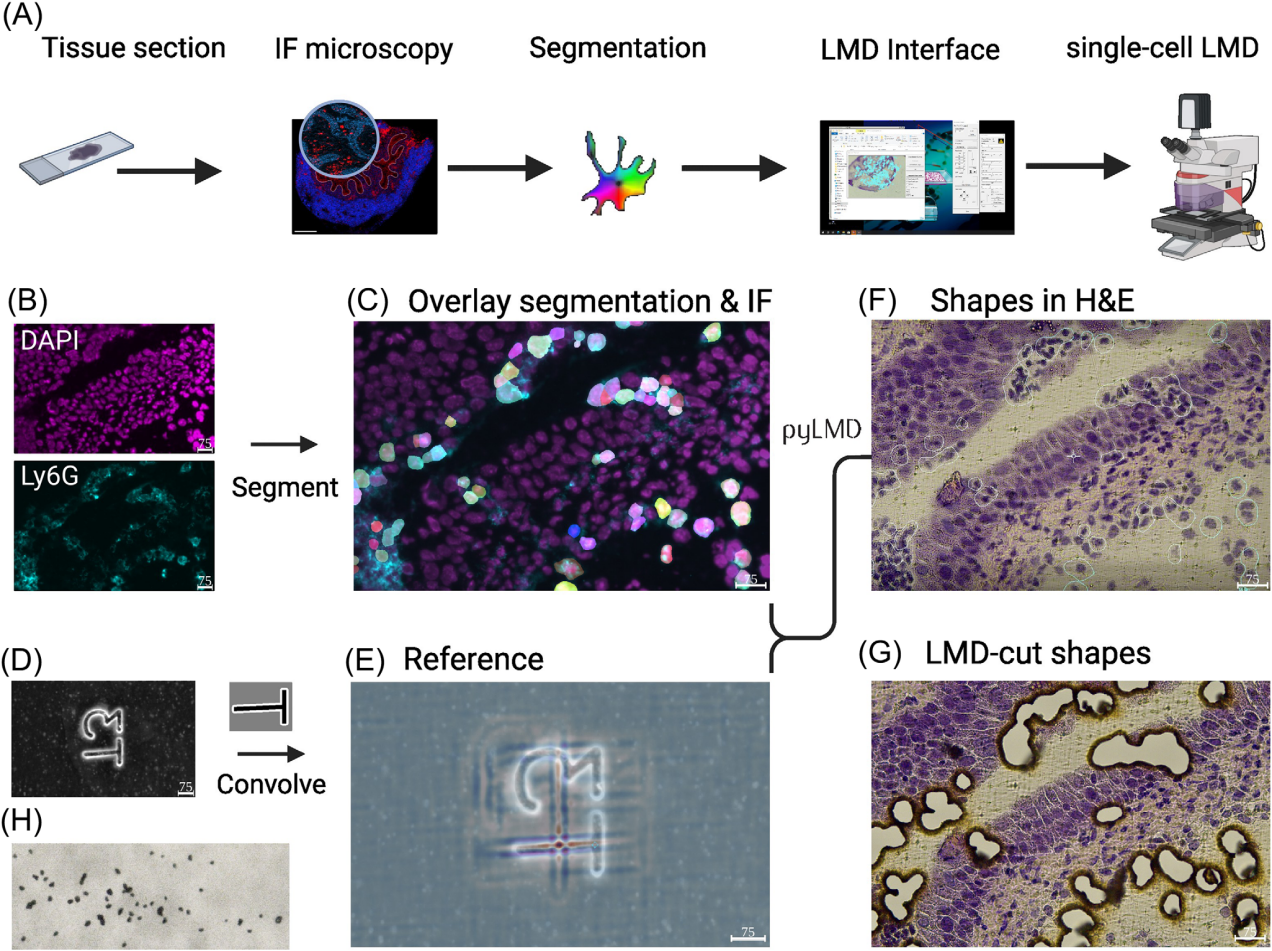
Workflow for Deep Visual Proteomics. (A) Schematic workflow for DVP. Figure was generated in BioRender. (B) Tissue sections of the murine urinary bladder were stained with fluorescently labelled reagents against DAPI (nucleus) and Ly6G (neutrophils). (C) Cell segmentation of neutrophils was performed using Cellpose‐SAM. (D) Fiducial markers on the tissue section for alignment of the digital images and physical slide using T‐shape convolution. (E) Results of the semi‐automated fiducial marker detection. (F) Transfer of the segmented images (C) and the fiducial markers (E) to the physical slide using pyLMD. Shapes were combined using pyLMD and prepared for laser capture microdissection. (G) Image of the physical slide on the LMD stage after LMD cutting was performed. (H) Microscopic validation of LMD‐cut neutrophil shapes in the LoBind Eppendorf tube. Scale bars are 75 pixels each with 1 pixel = 0.325 µm.

### 2.2 Segmentation and cellular boundary determination

Accurate segmentation of cells is essential for DVP. Boundary precision is especially critical as minor inaccuracies in defining cell contours propagate downstream, influencing which compartments are excised and ultimately altering readouts and analyses. Moreover, the segmentation of immune cells, such as neutrophils, is complicated by the complex cellular morphology and tissue microenvironments. Recent advances in generalised segmentation models such as Cellpose – Segment Anything Model (SAM) and related architectures have dramatically lowered the barrier to high‐quality segmentation across diverse tissue types.^10^, ^11^ These models offer zero‐shot generalisation to unseen datasets, while also supporting user‐guided fine‐tuning that allow to iteratively refine results and adapt models to specific tissues with minimal manual annotations. Transparent reporting of segmentation models, training datasets, and active learning strategies remains essential to ensure reproducibility and comparability across studies. Thus, we devised a simple‐to‐operate pipeline (Figure 1A) to extract single neutrophils from the murine urinary bladder in accordance with the FAIR principles. To this end, we stained tissue sections with Ly6G (neutrophils) and DAPI (cell nucleus) (Figure 1B). Cellpose‐SAM without fine‐tuning was subsequently used to segment neutrophils from fluorescent images (Figure 1C). As membrane associated Ly6G staining can broaden the apparent cellular boundary beyond the true physical contour of neutrophils, we increased the cell probability threshold of Cellpose‐SAM to 0.8. Manual inspection of the segmentation boundaries was used to validate the quality of the segmentation. Thus, using the automated cell segmentation algorithm, neutrophils were detected and segmented within the tissue section.

### 2.3 Instructions for interfacing with LMD instrument to excise neutrophils from tissue

Next, we aimed to laser‐micro‐dissect the segmented neutrophils using the cut function of the LMD instrument. Current workflows for interfacing with the LMD instrument often depend on proprietary software ecosystems, introducing barriers to interoperability and reproducibility. Mismatches between image analysis outputs and the LMD stage control system can result in mis‐targeting, tissue loss, or contamination. To address these challenges and reduce the risk of mis‐targeting, we established a standardised bioimage analysis and registration pipeline using an open‐access framework. After neutrophil segmentation with Cellpose‐SAM, the x/y coordinates of the digital images need to be aligned with the physical slide on the LMD stage. For this purpose, an automated detection of fiducial markers was established to reduce manual bias and alignment time. Thus three fiducial markers in the form of the stem and bar intersection of the letter ‘T’ (T1, T2, and T3) were burned into the tissue section using the LMD laser prior to acquiring the Ly6G‐ and DAPI‐stained images (Figure 1D). The points were arranged as a right‐angled triangle covering the segmented tissue areas, with each ‘T’ featuring a 180‐pixel stem, a 120‐pixel bar, a 15‐pixel width, and a slight 2.5° tilt. The mapping and selection of these fiducial markers are often performed manually, which can introduce user‐dependent variability during coordinate alignment. To automate this process, we have implemented an algorithm for automatic detection of these fiducial markers. As the autofluorescence inside of these “T” is reduced with a narrow bright surrounding halo, we constructed a structure element to match these features and performed fast Fourier transform (FFT) convolution to accurately determine the intersection of each ‘T’ stem and bar (Figure 1D and E). Manual inspection of the fiducial marks was used to validate the quality of the automated detection.

The fiducial markers along with the segmented cell shapes were exported as an XML file using pyLMD and subsequently imported into the LMD software where the fiducial markers were used to align the digital image with the physical slide. The potential cuts were checked using H&E staining (Figure 1F). Afterward, we proceeded with the cutting of the segmented neutrophils (Figure 1F), and the precision was validated in the H&E imaging (Figure 1G). Microscopic validation of LMD‐cut neutrophil shapes in the collection tube was performed by visual confirmation of successful capture and morphological integrity using the integrated LMD microscope (Figure 1H). Since the precision of excision with the LMD instrument is constrained by the optical resolution of the LMD system, tissue preparation quality, and laser parameters, a thorough description of the metadata is provided in the Material and Methods section. Thus, we demonstrate a pipeline for the alignment of the digital image with physical slide using a robust file format compatibility, coordinate system alignment through semi‐automated fiducial marker detection, and metadata synchronisation to ensure general applicability and reproducibility using neutrophil cutting as a use case.

## 3 CONCLUSION AND OUTLOOK

In summary, our study demonstrates a semi‐automated pipeline to segment microscopy images for laser microdissection of single cells from tissue sections. By systematically streamlining the workflow, we demonstrate that precise and reproducible excision of neutrophils is feasible even in structurally organised and heterogeneous tissues. While Cellpose‐SAM performs robustly for segmentation of individual cells, particularly in sparse or morphologically well‐defined populations such as neutrophils, segmentation accuracy may decrease in highly complex tissue architectures, densely packed cellular environments, or larger multicellular tissue regions. In such settings, additional model fine‐tuning, manual curation, or alternative segmentation strategies may be required. These methodological refinements are designed to reduce contamination risk and improve the fidelity with which computationally defined boundaries can be translated into physical microdissection paths and to reduce user‐dependent variability during image‐to‐stage alignment. Adaptation of the workflow to other tissues primarily requires adjustment of segmentation parameters, intensity thresholds, and potentially fine‐tuning of the segmentation model depending on marker distribution and tissue morphology. The XML export layer is modular and may be extended to support alternative LMD coordinate schemas.

Recent image‐to‐LMD interoperability tools such as ShapeUpLMD and OpenDVP/QuPath‐to‐LMD have advanced the field by enabling contour optimisation and export of annotated regions for laser microdissection.^12^, ^13^ In contrast, our workflow focuses on automated fiducial detection via FFT‐based template matching and integrates segmentation filtering, coordinate registration, and XML export into a unified Python‐based pipeline independent of proprietary image analysis software and manual annotation. This complements existing solutions by providing an end‐to‐end open framework for reproducible image‐to‐LMD interoperability. Collectively, these advances strengthen the DVP workflow and provide a robust framework for applying DVP to spatially resolved studies of immune cell function.

While qualitative validation demonstrated robust alignment of exported contours with target cells, future studies should systematically benchmark alignment precision, contamination rates, and cutting accuracy across diverse tissues and LMD platforms. Collectively, these advances strengthen the DVP workflow and provide a robust framework for applying DVP to spatially resolved studies of immune cell function. To support transparency, interoperability, and long‐term reuse, our workflow aligns with the FAIR principles through complementary public software and workflow archives. The source code is openly available in a version‐controlled GitHub repository, released under an Apache‐2.0 license, and archived as citable, versioned releases on Zenodo with persistent DOIs. The executable workflow is additionally registered on WorkflowHub, where it is described as a stable Jupyter‐based workflow with workflow‐level metadata and a persistent workflow DOI, supporting discovery and reuse through FAIR‐oriented workflow infrastructure. Interoperability is supported by standards‐based exchange of image‐derived contours and coordinates as XML files for import into Leica LMD software, while qualified references to related software components, including Cellpose‐SAM, pyLMD, Pixi‐managed dependencies, and the associated Zenodo dataset, document the computational environment and linked research objects. Reusability and reproducibility are further supported by an open license, version history, installation instructions, explicit dependency specification, configurable channel/parameter settings, example data, and detailed reporting of the experimental metadata and protocol.

Moreover, future directions may include the development of open access standards and interfaces for image‐to‐LMD communication, improved generalised models with user‐guided refinement for segmentation in complex tissues, and FAIR‐compliant pipelines linking imaging metadata with proteomic readouts. By addressing these challenges, DVP can evolve from a promising concept into a robust and automated tool for uncovering cellular diversity in health and disease.

## 4 METHODS

### 4.1 Animal studies

Six‐ to eight‐week‐old female C57BL/6J mice were used throughout the experiments. Animals were purchased from Charles River Laboratories and maintained under specific‐pathogen‐free conditions in the central animal facility at the University Hospital Essen. The mice were kept at 20–24°C and 45%–65% humidity in a 12‐h dark‐light cycle. They were housed in stable social groups and provided with species‐appropriate food and water, suitable bedding and nesting material as well as a shelter for rodents. The local review board (Bezirksregierung Köln, Landesamt für Natur, Umwelt und Verbraucherschutz NRW in Recklinghausen, Germany) approved the animal experiments.

### 4.2 Urinary tract infection model

UPEC strain 536 (O6:K15:H31) was cultured for 3 h at 37°C in LB medium (X964, Carl Roth GmbH). The bacteria were harvested by centrifugation at 1500 × *g* for 20 min and then the OD(600) was measured. The bacteria were resuspended at a concentration of 10^10^ bacteria/mL in sterile PBS. A mixture of ketamine (402581.00.00, bela‐pharm) and xylazine (3100265.00.00, Serumwerk Bernburg AG) 80/10 mg/kg body weight in 150 µL PBS (18912‐014, Life Technologies) was injected intraperitoneally to anesthetise the mice. The infection of the animals was induced by transurethral inoculation of 5 × 10^8^ UPEC in 0.05 mL PBS using a soft polyethylene catheter (G944‐A01, icumedical) and mice were sacrificed one day after infection.

### 4.3 Workflow

1. Burn fiducial T‐marks into tissue section using LMD laser
2. Acquire fluorescence microscopy images
3. Perform Cellpose‐SAM segmentation
4. Apply intensity and overlap filtering
5. Detect fiducials via FFT‐based template matching
6. Determine and export contours and coordinates via py‐lmd as XML
7. Import XML into Leica LMD software
8. Align fiducials and perform excision
9. Validate excision microscopically

### 4.4 Immunofluorescence microscopy

UPEC‐infected urinary bladder was fresh frozen in 2‐methylbutane (740379, Carl Roth GmbH) on dry ice. Bladder tissue was cut into 8 µm thick sections at −20°C using a cryostat (Leica CM 1950, Leica) and mounted on PPS frame slide, 4 µm (#11600294, Leica). Sections were fixed for 5 min on ice using 4% formalin (4235, Carl Roth GmbH). Sections were washed for 30 sec with PBS and then incubated with directly conjugated antibody against Ly6G (127610, BioLegend, 1:200) for 1 h at RT in a humidified chamber. Sections were washed for 30 sec three times with PBS, before nuclei were counterstained using DAPI (D1306, Life Technologies, 1:5000). After a short wash with distilled water, a coverslip (9.161060, Oehmen Labortechnik) was mounted with 50% glycerol (56‐81‐5, Thermo Fisher Scientific). After imaging, the coverslip was removed washing the slides for 10 min in MilliQ water and air dried until laser microdissection.

### 4.5 Imaging and LMD

Immunofluorescence images of urinary bladder sections were acquired using an Axio Scan Z.1 system (Zeiss). To lower electrostatics and ensure best possible cutting results, the tissue was stained with H&E fast staining kit (9194, Carl Roth) according to the manufacturer´s protocol, shortly dipped in 70% ethanol (1HP8, Carl Roth GmbH) and air dried before cutting. LMD (LMD7, Leica) was performed with the Leica LMD7 system and XML files were then imported into Leica LMD software (v8.4.0.8759) for precise alignment and microdissection. After aligning the fiducial marker and mask coordinates to the Leica LMD7 laser microdissector at 40× magnification, the laser microdissection of the cells of interest was performed under the following parameters: middle pulse cutting method was selected, laser power was set to 20 (use the lowest power possible to avoid heating up the slide), aperture was set to one, cutting speed was set to 4, middle pulse count was set to two, final pulse was set to one, head current was at 70%, and pulse frequency was set to 2100 Hz. The Leica Laser Microdissection software was utilised for automated excision and collection of contours. Cells were collected into the caps of 0.5 mL Protein LoBind tubes (0030108094, Eppendorf). Then, 60 µL of 100% Acetonitrile (01207801, Biosolve) were added, and samples were centrifuged for 5 min at 13,000 × *g* at RT to concentrate tissue at the bottom of the tube, followed by vacuum evaporation until completely dried. If cutting is performed at changing temperatures, we recommend regularly reimporting shapes, realigning fiducial markers, and recalibrating the laser to maintain targeting precision. To avoid cross‐contamination of the surrounding tissue, the thickness of the LMD cut and the size of the segmented cell areas should not exceed half of the diameter of the cells to be cut.

### 4.6 Cell segmentation and preparation for LMD

Segmentation masks of neutrophils were generated on contrast‐stretched [0.1, 99.9] and normalised [0, 1] DAPI and Ly6G stains using Cellpose‐SAM (v4.0.7) using a cell probability threshold (cellprob_threshold) of 0.8 to account for broadened membrane marker.^10^ Segmented masks were filtered to retain cells with a mean Ly6G intensity ≥ 0.35 and ≥ 95% spatial overlap with Ly6G‐positive pixels (Ly6G > 0.2). The selection of these thresholds was based on visual inspection of three representative image fields, testing multiple intensity cut‐offs to identify criteria that reliably distinguished neutrophils from surrounding cells. A mean Ly6G intensity threshold of 0.35 removed approximately 95% of non‐neutrophil segments. Stricter thresholds resulted in the unintended exclusion of isolated neutrophils, which typically display a ring‐shaped Ly6G distribution with lower signal intensities in the nuclear region. The second criterion (≥ 95% of pixels > 0.2) ensured a minimum spatial signal homogeneity and enabled the exclusion of larger, Ly6G‐inhomogeneous cells arising within neutrophil clusters. The resulting segmentations were subsequently reviewed jointly by two independent investigators and deemed qualitatively robust. Fiducial mark detection was performed on the autofluorescence of the images. They were normalised to [0, 1], intensity‐inverted, and contrast‐enhanced using adaptive histogram equalisation (equalise_adapthist, scikit‐image v0.25.2) before a second inverting.^14^ A T‐shaped template was matched to the processed image by FFT convolution (fftconvolve, SciPy v1.16.3)^15^; the intersection of each T's bar and stem was subsequently extracted from the top three peaks determined using peak_local_max (scikit‐image) to serve as the fiducial markers for LMD. Filtered cell contours combined with the fiducial marks were exported as an XML file with py‐lmd (v1.0.0) and forwarded to the LMD.^16^

## Data Availability

The entire workflow has been released at Github https://github.com/Immunodynamics‐Engel‐Lab/lmd‐imageanalysis, at Zenodo https://doi.org/10.5281/zenodo.20067089 and on WorkflowHub https://doi.org/10.48546/workflowhub.workflow.2168.1. Data are available at Zenodo: https://doi.org/10.5281/zenodo.17792441.
